# Common Laboratory Parameters Are Useful for Screening for Alcohol Use Disorder: Designing a Predictive Model Using Machine Learning

**DOI:** 10.3390/jcm11072061

**Published:** 2022-04-06

**Authors:** Juana Pinar-Sanchez, Pablo Bermejo López, Julián Solís García Del Pozo, Jose Redondo-Ruiz, Laura Navarro Casado, Fernando Andres-Pretel, María Luisa Celorrio Bustillo, Mercedes Esparcia Moreno, Santiago García Ruiz, Jose Javier Solera Santos, Beatriz Navarro Bravo

**Affiliations:** 1 Department of Internal Medicine, Jose Maria Morales Meseguer University General Hospital, 30008 Murcia, Spain; juanipinarsanchez@gmail.com; 2 Computer Science Department, Universidad de Castilla-La Mancha, 02071 Albacete, Spain; pablo.bermejo@uclm.es; 3 Unit of Infectious Diseases, Department of Internal Medicine, University General Hospital of Albacete, 02006 Albacete, Spain; 4 Unit and Gerodontology, Department of Dermatology, Stomatology, Radiology and Physical Medicine, Special Care Dentistry, Jose Maria Morales Meseguer University General Hospital, Faculty of Medicine, University of Murcia, 30008 Murcia, Spain; redondruiz@gmail.com; 5 Department of Biochemistry, University General Hospital of Albacete, 02006 Albacete, Spain; lnavarro@sescam.jccm.es; 6 Clinical Research Support Unit, National Paraplegics Hospital of Toledo Foundation, 45004 Toledo, Spain; fandresp@sescam.jccm.es; 7 Department of Mental Health, Addictive Conducts Unit Care in Albacete, 02005 Albacete, Spain; mlcelorrio@gmail.com (M.L.C.B.); esparciamoreno@gmail.com (M.E.M.); 8 Blood Donation Center from Albacete and Cuenca, Department of Hematology, University General Hospital of Albacete, 02006 Albacete, Spain; garciaruizsantiago@gmail.com; 9 Department of Internal Medicine, University General Hospital of Albacete, 02006 Albacete, Spain; solera53@gmail.com; 10 Department of Psychology, Faculty of Medicine, Universidad de Castilla-La Mancha, 02008 Albacete, Spain

**Keywords:** data science, alcohol-related disorders, screening, laboratory diagnosis, machine learning

## Abstract

The diagnosis of alcohol use disorder (AUD) remains a difficult challenge, and some patients may not be adequately diagnosed. This study aims to identify an optimum combination of laboratory markers to detect alcohol consumption, using data science. An analytical observational study was conducted with 337 subjects (253 men and 83 women, with a mean age of 44 years (10.61 Standard Deviation (SD)). The first group included 204 participants being treated in the Addictive Behaviors Unit (ABU) from Albacete (Spain). They met the diagnostic criteria for AUD specified in the Diagnostic and Statistical Manual of mental disorders fifth edition (DSM-5). The second group included 133 blood donors (people with no risk of AUD), recruited by cross-section. All participants were also divided in two groups according to the WHO classification for risk of alcohol consumption in Spain, that is, males drinking more than 28 standard drink units (SDUs) or women drinking more than 17 SDUs. Medical history and laboratory markers were selected from our hospital’s database. A correlation between alterations in laboratory markers and the amount of alcohol consumed was established. We then created three predicted models (with logistic regression, classification tree, and Bayesian network) to detect risk of alcohol consumption by using laboratory markers as predictive features. For the execution of the selection of variables and the creation and validation of predictive models, two tools were used: the scikit-learn library for Python, and the Weka application. The logistic regression model provided a maximum AUD prediction accuracy of 85.07%. Secondly, the classification tree provided a lower accuracy of 79.4%, but easier interpretation. Finally, the Naive Bayes network had an accuracy of 87.46%. The combination of several common biochemical markers and the use of data science can enhance detection of AUD, helping to prevent future medical complications derived from AUD.

## 1. Introduction

Alcohol dependence is a frequent medical problem in a wide variety of clinical settings and requires attention to reduce medical complications, establish appropriate treatments, and minimize the use of healthcare resources [1]. Alcohol abuse has harmful effects that can cause or contribute to multiple diseases [2] and even increase the risk of cancer [3,4] and deaths [5]. 

Alcohol has also increased traffic accidents, trauma [6], absenteeism [7] at the workplace and school [8], job loss, violent behavior, and legal [9], family, economic, mental [10], and social problems [5]. Despite the significant benefits of alcohol abuse treatments [11], general practitioners only manage to diagnose a third of these patients [12]. Self-assessment questionnaires (for example, AUDIT (Alcohol Use Disorders Identification Test), CAGE (Cuttingdown, Annoyed, Guilt, Eyeopen), MAST (Michigan Alcohol Screening Test)) are typically used [13] to detect alcohol use disorder [14], but these can be misleading if patients are reluctant to reveal their patterns of alcohol use [15]. 

Laboratory markers can corroborate clinical suspicion of alcohol abuse and facilitate patient monitoring and compliance with the recommendations. For instance, alcohol biomarkers are physiological indicators of alcohol exposure and can help detect AUD [16]. These biomarkers are more useful when used together with other information such as medical history. Biomarkers can be classified as direct and indirect [17]. Direct markers include detection of alcohol or its metabolites such as ethyl glucuronide, derivatives of acetaldehyde, phosphatidyl ethanol, and ethyl esters of fatty acids. These biomarkers have high specificity but low sensitivity due to their short plasma half-life. However, ethylglucuronide can be detected in urine up to 5 days and can inform us of chronic consumption by measuring it in the hair [18]. Regarding acetaldehyde derivatives, studies have described that they can be used as alcohol use markers in women [19]. Furthermore, the quantification by anti-adduct autoantibodies has been shown to have clinical value, differentiating abstinent users from alcohol users [20,21].

Indirect markers such as aspartate-aminotransferase (GOT/AST), alanine-aminotransferase (GPT/ALT), gamma-glutamyltransferase (GGT), erythrocyte mean cell volume (MCV), uric acid, HDL-cholesterol, triglycerides, cholesteryl ester transfer protein (CETP), and total serum sialic acid (TSA) [22] help identify excessive alcohol use by detecting its toxic effects. They are inexpensive and widely available, but they have the disadvantage of low sensitivity and unspecificity [17]. In addition, the alcohol-induced thrombocytopenia can be the first signal of alcohol use disorder, also being a prognostic factor in the development of alcohol withdrawal syndrome and bleeding [23]. New biomarkers, more sensitive and specific, have been investigated in the last 30 years, with carbohydrate-deficient transferrin (CDT) being the most widely adopted [9,10]. An emerging biomarker is N-acetyl-Beta-Hexosaminidase (Beta-Hex), which could be used for acute alcohol intoxication, but has been used in preclinical research with rats, as well as some patients with Tay–Sachs disease with a diagnostic test. Therefore, alcohol researchers need to investigate more for using beta-Hex as a diagnostic tool [22]. 

Some studies have used parallel testing, combining two or more laboratory tests to identify alcohol abuse with increased diagnostic accuracy [24,25,26]. For example, CDT and GGT combinations improve the diagnostic yield of any of these markers used alone [27,28]. Similarly, parallel tests with CDT, GGT, and MCV improve detection in women [29], while CDT and the AUDIT help detect alcohol use disorders in a regular workplace [30]. 

An alternative strategy to specifically using alcohol biomarkers is the use of multiple routine laboratory tests and statistical methods [25,26,31]. For instance, the Early Detection of Alcohol Consumption (EDAC) method uses a linear discriminant function [32] that analyzes 10 routine laboratory tests to generate a score for each subject. Each score and its associated probability value translate into the likelihood that the individual has a specific consumption pattern [25]. Ten laboratory measurements are included in the final regression equation: chloride, sodium, direct-to-total bilirubin ratio, blood urea nitrogen, high-density lipoprotein, monocyte count, phosphorus, platelets, aspartate aminotransferase, and mean corpuscular hemoglobin (HCM). In the validation data for this model, 98% of the 161 heavy drinkers and 95% of the 42 mild drinkers were correctly identified. Widespread adoption of these statistical methods is limited due to limited availability of the software and hardware packages required. 

In this study, we describe a laboratory model to predict patients’ risk group of alcohol consumption using discriminant analysis. The laboratory parameters used are routinely included in clinical consultations. Hence, our study can facilitate the early and inexpensive diagnosis of alcohol use, as well as better treatment monitoring. 

We hypothesize that a combination of routine biochemical markers can predict the probability of alcohol consumption and improve the results obtained with standardized alcohol use questionnaires in patients with unknown risk [33] of alcohol consumption. 

## 2. Materials and Methods

This study is a retrospective observational study in which the relationship between laboratory test results and patient alcohol consumption was analyzed. Participants were recruited from two different groups: patient from Addictive Behaviors Unit (ABU) and blood donors. 

The ABU group (retrospectively recruited) included patients according to the following criteria: (1) Patients who started treatment at ABU from Albacete in the detoxification phase due to alcohol consumption during 2013–2015 (selected retrospectively, from June 2014), at any age. (2) Patients who meet the diagnostic criteria for alcohol use disorder (AUD) specified in the Diagnostic and Statistical Manual of mental disorders fifth edition (DSM-5); they were correctly diagnosed by a mental health expert doctor. (3) Patient’s laboratory test results at the start of treatment in the ABU were available (up to 1 month prior or 90 days after). This information was extracted by consulting the ABU medical records and the laboratories database (in this case, for laboratory test results). Data were pseudo-anonymized by the research team, and participants could only be identified by their assigned code. The exclusion criteria were: (1) Serious mental illness incompatible with participation in the study (with consent). (2) Not having laboratory test results when they started in ABU treatment (up to 1 month before or 90 days after). (3) Patients who followed treatment with any drug that could disturb the blood parameters, from which the prediction was to be made.

The other group (recruited by cross-section) was obtained from a sample of blood donors from Albacete. Serum samples from these donors were used to analyze the same analytical parameters collected (biochemical, hematological, and coagulation) in the ABU participants. The Spanish version of the 10-item AUDIT (Alcohol Use Disorder Identification Test) was administered as a written questionnaire to assess alcohol consumption in these participants, ruling out donors with risky alcohol consumption (AUDIT score > 8 in men or >6 in women) [34]. AUDIT assesses the frequency and quantity of drinking (questions 1–3) and alcohol-related harm (questions 4–10). The study’s objectives were explained to these participants and written informed consents collected. 

All the blood parameters studied were: albumin, amylase, activated partial thromboplastin time, uric acid, basophils, basophils_percent, direct bilirubin, indirect bilirubin, total bilirubin, calcium, mean corpuscular hemoglobin concentration, creatine kinase, chlorine, coagulation, cholesterol, creatinine, eosinophils, eosinophils_percent, red blood cells, alkaline phosphatase, ferritin, fibrinogen C, gamma glutamyl transferase, globulins, glucose, aspartate aminotransferase, alanine aminotransferase, hemoglobin, mean corpuscular hemoglobin, hematocrit, high-density lipoprotein cholesterol, red blood cells distribution width, platelet distribution width, International Normalized Ratio, potassium, lactate dehydrogenase, low-density lipoprotein cholesterol, white blood cells, lymphocytes, lymphocytes_percent, large unstained cells percent, large unstained cells, monocytes, monocytes_percent, myeloperoxidase index, neutrophils, neutrophils_percent, phosphorus, C-reactive protein, platelets, total proteins, tryglicerides, transferrin, urea, mean corpuscular volume, mean platelet volume, and sodium.

**Figure 1:**
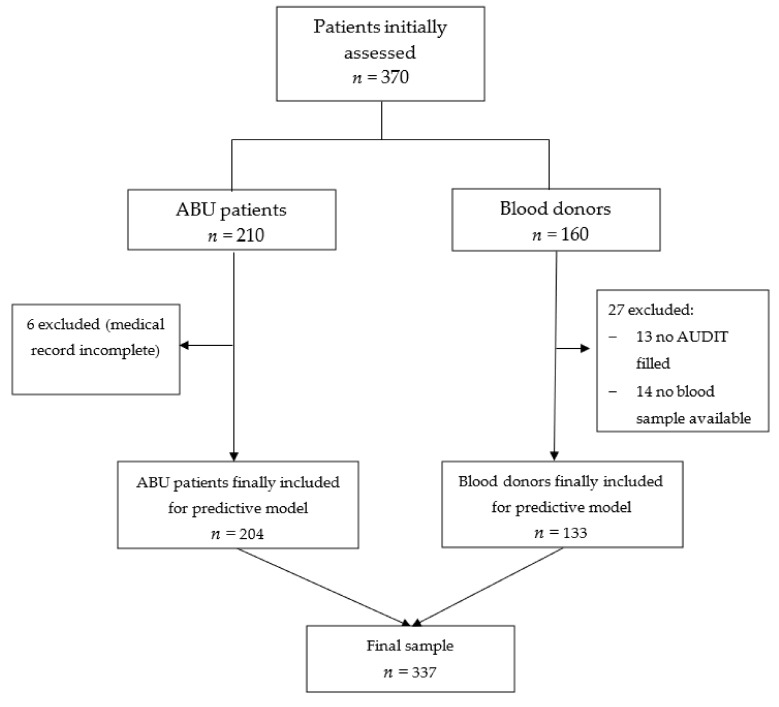
Flow chart of the study.

A total of 337 participants were included in this study after exclusions (due to incomplete data), including 204 in the ABU group and 133 participants in the blood donor group. You can see the flow chart of the study in Figure 1.

Our sample had 253 male participants (75.3%) and 83 female participants (24.7%). The age of the participants ranged from 15 to 78. The mean age in the ABU participants was 45.5 years (Standard Deviation: SD = 10.06), and the mean age in the blood donor participants was 42.5 (SD = 10.89).

We obtained some medical history in the ABU group: age, sex, address (if city or village), study level, marital status, age that alcohol consumption started, and consumption of other drugs (nicotine, cocaine…). The same variables were obtained from the blood donor group, except the last two ones.

For the predictive analysis, participants were classified as alcohol consumption according to their average weekly consumption and gender, as per the WHO classification. More specifically, a value of >28 SDUs (Standard Drink Unit) was used for men and >17 SDUs for women [35]. Alcohol consumption was measured in Standard Drink Units (SDUs), using the equivalence of 10 grams of pure alcohol = 1 SDU [36]. Then, we conducted a bivariate statistical analysis, correlating the lab test parameters analyzed with the number of SDUs consumed. Subsequently, three predictive models were created, using different approaches: logistic regression, classification tree, and Bayesian network. 

Complete participant data and variables’ definitions can be found in the supplementary information material. Supplementary Table S1: Variables’ definition used in the predictive model. 

We depurated the database, and the statistical analysis was carried out with SPSS program 25.0.0. Values were expressed as mean (SD), and results were considered to be statistically significant with a *p* value < 0.05. For the execution of the selection of variables and the creation and validation of predictive models, 2 tools were used: the scikit-learn library for Python and the Weka application.

Scikit-learn with the Python [37] programming language is the most widely used data science tool today to perform predictive model building and processing tasks. Normally, it is only usable by people with programming knowledge such as computer engineers or mathematicians. In cases such as this article, the medical doctors contacted an expert in data science to explain and guide them in the process of discovering useful predictive models, with said data science expert taking charge of the programming part, always with the guidance and supervision of the clinician. Supplementary Information Figure S1 shows an extract from the notebook generated with the predictive analysis with Scikit-learn, used for the logistic regression model as well as for the classification tree model.

It should be noted that both the database used and the code generated for this part of the predictive analysis were shared from an institutional repository of the University of Castilla La Mancha (UCLM), specifically in the section corresponding to the SIMD research group (Intelligent Systems and Data Mining), belonging to the contacted machine learning expert. The repository link is: https://github.com/UCLM-SIMD/alcohol_risk_prediction (last access and update 25 October 2021).

Weka [38] is an application developed by the University of Waikato, in New Zealand. This tool allows any trained user to perform various tasks of data science, such as variable selection, normalization, imputation of missing values, and predictive modeling. This is performed relatively easily thanks to its intuitive interface, as can be seen in Figure S2, in the Supplementary Information. 

The logistic regression model used automatic variables selection by Incremental Wrapper Subset Selection with replacement (IWSSr) [24]. In IWSSr, even if a variable has been selected, it may cease to be important in the presence of a new candidate variable. When performing a common forward search, the algorithm selects features according to their relevance for the prediction. However, it is possible that a previous relevant feature is discarded if other, more relevant features are included. For instance, if a patient shows symptoms of sinusitis or they feel cold, these might be good variables to predict the probability of having the flu. However, if we also learn that they have high fever, sinusitis is no longer an important feature to predict flu. This property of IWSSr becomes particularly relevant when the cost associated with each feature is considered. That is, if the discarded feature is more expensive than the new one (e.g., in our case, ferritin is more expensive than creatinine), our IWSSr method can lead to improvements both in terms of economic cost and algorithmic complexity. 

The classification tree (the second model created) creates easily interpretable models, creating a path from its root node through the value of the case to be classified in the selected variables until it reaches a leaf node, with an associated classification result. The third model-building process captures variables dependencies and builds the simplest and efficient model: a Naive Bayes network. Previously, the variables were discretized following Fayyad and Irani’s method based on entropy (uncertainty) concerning the class (risk).

All the predictive models created were validated with the LOO (leave-one-out validation) method. LOO is a good validation scheme when the number of samples is not large. Give a database with N samples, LOO trains the predictive classifier with N-1 samples and then tests its prediction on the Nth sample, and this is repeated N times, each time with a different test sample. Then, metrics are computed based on the total positives (TP), total negatives (TN), false positives (FP), and false negatives (FN) performed by the classifier during the N train-test steps.

As missing values in the variables were input, the mean of the corresponding analytical variable was applied. This was applied in the logistic regression and classification tree models. Data were normalized to mean 0 and variance 1 for the logistic regression models. The preprocessed imputation of missing values and normalization were always applied to the corresponding training set for each split in the LOO validation. 

## 3. Results

The distribution by sex and age is similar in both groups. In the ABU group, the sample was represented by 78.4% men and 21.6% women. The blood donor group, and therefore participants without risk of exposure to alcohol, was made up of 133 participants, distributed as 70.7% men and 29.3% women.

The mean ages in both groups were comparable, 45 years old for participants in the ABU group, with a standard deviation of 10.05, and a mean of 41 years old for participants in the blood donor group, with a standard deviation of 10.89. They had age ranges of (15–78) and (18–66), respectively, with the variances being homogeneous and reaching statistical significance (*p* < 0.000).

Regarding the address, 72.7% lived in a town and 27.3% lived in a city.

**Figure 2:**
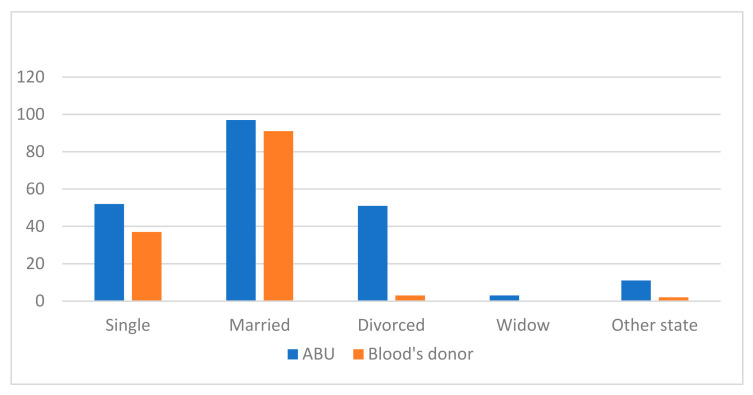
Comparison of marital status for both groups, ABU and blood donors.

The marital status of both groups can be seen in Figure 2.

The marital status of the ABU participants was: 52 single (25.5%), 97 married or in a stable union (47.5%), 51 separated or divorced (25%), 3 widowed (1.5%). Within other marital statuses, 5 lived with a partner, 1 had a stable partner but did not live with them, and 5 lived with a common-law partner.

The marital status of the blood donor participants was: 37 single (27.8%), 91 married or in a stable union (68.4%), 3 separated or divorced (2.3%), 2 participants had other marital status (1.5%) (one lived as a couple and the other as a de facto couple).

**Figure 3:**
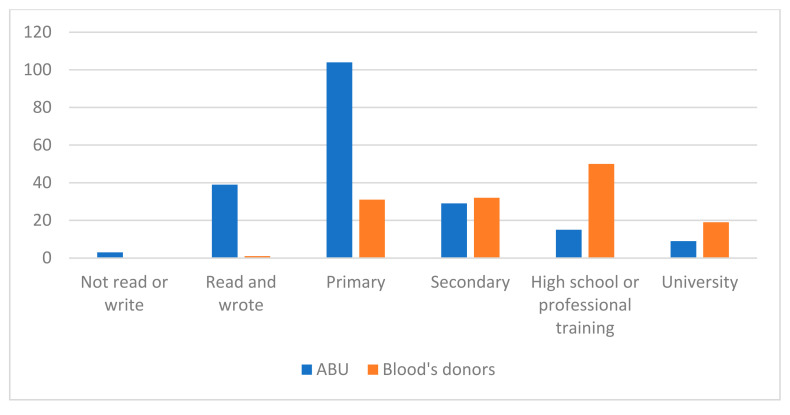
Comparison of the study level for both groups (ABU and blood donors).

We also considered it important to observe the study level comparing both groups, which is represented in Figure 3.

The study level that predominated in users of the ABU was primary studies with 104 participants (51%), 39 participants read and wrote but had no studies (19.1%), 29 participants had secondary studies (14.2%), 15 participants had completed high school or professional training courses (7.4%), 9 had completed university studies (4.4%), and 3 participants did not read or write (1.5%). 

The predominant study level of the blood donors in our sample was high school and/or professional training (50 blood donors, 37.6%), followed by 32 blood donors with secondary education (24.1%), 31 blood donors with primary education (23.3%), 19 blood donors with university studies (14.3%), and only 1 blood donor (0.8%) read and write but did not have studies.

In our sample, the age of onset of alcohol consumption had a mean of 17 years old, with a standard deviation of 7.18 and a standard error of 0.507.

Regarding the consumption of other drugs, the ABU’s participants had the following data recorded: tobacco, 135 participants (66.18); cannabis, 38 participants (18.62%), cocaine, 30 participants (14.7%); heroin, 5 participants (2.45%), benzodiazepines, 2 participants; crystal meth, 2 participants; tripis or LSD, 2 participants (1% each one); and Popper and amphetamine, 1 participant each (0.5%).

However, all heroin users reported quitting at the time of the interview, and reported previous use but not current use.

The next section summarizes the main results obtained for the three predictive models created (with logistic regression, classification tree, and Naïve Bayes). Complete data can be found in Supplementary Information.

Supplementary Information (online resource) Table S2 shows the correlation of different lab parameters with the binomial variable alcohol consumption of risk/no risk. S 2.1 all participants, S 2.2 men, and S 2.3 women. 

Supplementary Information (online resource) Figure S3 shows the correlation of lab parameters with consumption (S 3.1 all participants, S 3.2 men, and S 3.3 women). Supplementary Information (online resource) Figure S4 shows the rate of participants with risk of alcohol consumption. 

### 3.1. Logistic Regression

We tested three logistic regression models using different subsets of variables, and an example of manual use of these models can be found in Supplementary Material (Example S1). These models were created according to the results of our bivariable analysis. 

Table 1 shows the coefficients calculated for all the variables used, together with their confidence interval and their *p* values.

The first logistic regression model resulted in an accuracy of 83.9%, a sensitivity of 84.4%, and a specificity of 83.3%. Its positive predictive value was 84.4%, and the negative predictive value was 83.3%. 

Our statistical analysis revealed fifteen statistically significant variables within the model: mean corpuscular hemoglobin concentration, chlorine, eosinophils, alkaline phosphatase, ferritin, mean corpuscular hemoglobin, red blood cells distribution width, potassium, white blood cells, lymphocytes, monocytes, neutrophils, urea, mean corpuscular volume, and sodium. Therefore, we created a model using only these significant variables. The second simplified model resulted in an accuracy of 85.1%, a sensitivity of 86.7%, and a specificity of 85.4%. Its positive predictive value was 84.8%, and the negative predictive value was 85.4%. 

Finally, an automatic variable selection process using IWSSr was tested, which can be seen in Table 2.

This resulted in some variables being discarded (one of them was ferritin (more expensive)), resulting in a model with only 8 variables: mean corpuscular hemoglobin, gamma-glutamyl transferase, red blood cells distribution width, creatinine, total bilirubin, mean platelet volume, large unstained cells, and high-density lipoprotein cholesterol. This model provided very similar performance to the prior models, with an accuracy of 84.8%, a sensitivity of 83.8%, and a specificity of 83.2%. Its positive predictive value was 86.3%, and the negative predictive value was 83.2%. 

It is remarkable that there were 14 participants followed in the ABU and that they were classified as no risk for the predicted model, due to the number of SDUs declared in their consumption. With the result they obtained, they were considered positive by the model, and when calculating the sensitivity and specificity, they were by both considered as false positives among the 23 total false positives.

### 3.2. Classification Tree

**Figure 4:**
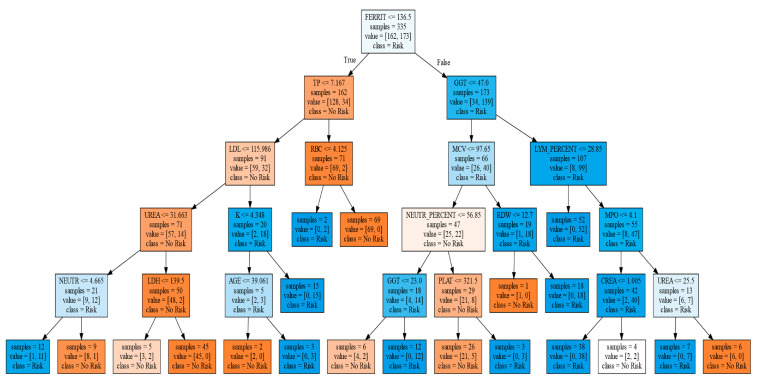
Classification tree. In that figure, the square brackets are not bibliographic references. It shows the generated tree, whose initial root node is with the variable ferritin, and depending on whether its value is greater or less than 136.5, it continues with total proteins (TP), or gamma-glutamyl transferase (GGT), and later, depending on the value of these, it continues toward different variables, to finally reach the different sheets, in the lower part, that predict whether the participant is at risk or not at risk, with respect to risky alcohol consumption.

Figure 4 shows the final classification tree produced. Although this presented a somewhat lower predictive accuracy than the logistic regression, classification trees are easier to interpret, as well as capable of explaining their inference process. Our classification tree resulted in an accuracy of 79.4%, a sensitivity of 78%, and a specificity of 77.5%. Its positive predictive value was 81.3%, and the negative predictive value was 77.5%.

It is worth noting that there were 20 participants followed in the ABU and that they were classified as nonrisk due to the number of SDUs declared in their consumption, they were considered as positive by the classification tree, and when calculating the sensitivity and specificity, they were therefore considered as false positives. Furthermore, among the 31 participants classified as false positives in the classification tree and 23 in the logistic regression (IWSSr), there were 14 coincidences.

### 3.3. Bayesian Network

**Figure 5:**
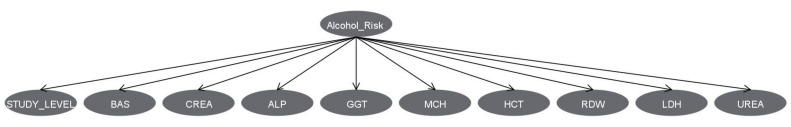
Prediction of alcohol use by 9 biomarkers and the study level using machine learning, obtained by a Bayesian network. BAS = Basophils, CREA = Creatinine, ALP = Alkaline phosphatase, GGT = Gamma glutamyl transferase, MCH = Mean corpuscular haemoglobin, HCT = Hematocrit, RDW = Red blood cells distribution width, LDH = Lactate Dehydrogenase.

This network, evaluated with all the variables or the subset resulting from an automatic variable selection process, gave its best results when using the 10 variables selected through the IWSS algorithm, starting with a subset without ferritin, as was the case with the logistic regression. The selected variables were: study level, basophils, creatinine, alkaline phosphatase, gamma-glutamyl transferase, mean corpuscular haemoglobin, hematocrit, red blood cells distribution width, lactate dehydrogenase, and urea. The accuracy was 87.5%, sensitivity was 88.3%, specificity was 86.6%, positive predictive value was 88.3%, and negative predictive value was 86.6%. The network created is shown in Figure 5, whose nodes contain the probability tables for risk and the discretized variables shown in Table 3. An interpretation example for this table is: given a patient at “risk” (according to the cut-off pattern used in this study), their probability of having a GGT value > 46.5 is 42.8%. If you are a “no risk” patient, only 7%. The Supplementary Material (Example S2) shows an example of prediction by using the Bayesian network. 

As in the other methods used, the Bayesian network classifies as positive (risk drinkers) some participants followed in the ABU who would belong to the group of no risk by the declared SDUs. Specifically, of the 20 false positives in the sample classified by this method, 14 were participants followed up at the ABU and initially classified as not at risk.

**Figure 6:**
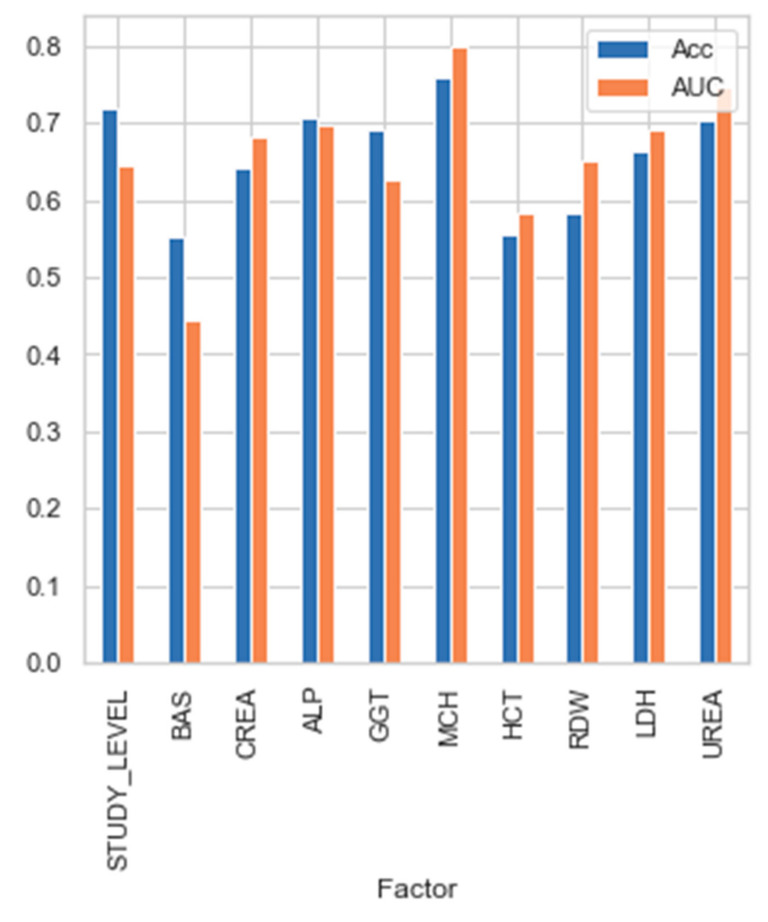
Predictive strength of each parameter selected in the Bayesian network.

Figure 6 shows the predictive strength of each selected parameter, as measured individually with the Bayesian network classifier. 

In terms of accuracy, we can see that none of them present such a good value as all the factors together. That is, while selected factors were not capable of providing predictive power individually (ranging from 55% to 75%), when their interaction was used to feed the classifiers, the accuracy improved greatly up to 87.5%. 

## 4. Discussion

This study used three methods to predict alcohol use disorder with a combination of biomarkers and medical information using machine learning and data science. As far as we know, this is one of the few studies to compare the discrimination accuracy of 48 combined biomarkers at the same time. In total, 52 variables were tested (48 numerical variables and 4 categorical variables) in the predictive model for detecting alcohol use. 

There is also evidence of the usefulness of some biomarkers individually, for example, the relationship between the lower platelet counts (<119 k/mL) and higher risk for complicated alcohol withdrawal syndrome [39]; however, our best predicted model using the Bayesian network did not select the platelet count with the best variables for combining. Regardless, this parameter was selected in the classification tree, just as the mean platelet volume was necessary for the prediction by logistic regression with IWSSr. 

Another study [40], similar to ours, used machine learning and combined psychological variables, substance use history, demographics variables, family history, and three laboratory biomarkers (thyroid stimulating hormone, hematocrit, and hemoglobin A1C percentage).

Some authors used machine learning in the prediction of AUD, with other features, such as electrophysiological (electroencephalogram coherence), psycho-social genetic information [41], and multimodal biomarkers; another study tested if risk factors for alcohol use disorders (family history, male sex, impulsivity, low level of response to alcohol) predicted the rate of binging throughout the alcohol self-intravenous administration session [42]. In the study of [43], they tested 38 features (sex and age), history of smoking, and some biomarkers (chemistry, liver function, hematology, and lipids). 

A systematic literature review by Ebrahimi et al. described the more important studies for AUD prediction using machine learning in the last 10 years. It concluded that several features were used for prediction, such as demographics, drinking behavior, family history, and electronic health records, but the lack of deep learning techniques for predicting AUD is evident and they suggested, as future research, challenges for the prediction of AUD [44].

Choosing a combination of variables can improve the predictive value of the variables separately. As can be seen in Figure 6, this clearly proves that predictive features should not be selected by evaluating them in a univariate manner, but multivariate evaluation is most advisable. The parameter predictive power individually is not so high (ranging from 55 to 75%), but when the parameters interact, the accuracy improves greatly up to 87.5%.

Our study selected the optimum combination of biomarkers as objective data by three different types of predicted models to overcome the weakness of self-assessment questionnaires, where people can deny or minimize the quantity or frequency of their patterns of alcohol use [45]. Our tool had a sensitivity from 78% with the classification tree to 88.3% with the Bayesian network, and a specificity from 77.5% with the classification tree to 86.6% with Bayesian network. Although the logistic regression’s accuracy was good, it did not fit the data used excessively well (R^2^ = 67%). The classification tree, despite its low accuracy (79.4%), had the advantage of interpretability. Our three predicted models (Logistic Regression (IWSSr), Classification Tree, Bayesian network) had positive predicted values of 86.3%, 81.3%, and 88.3% and negative predicted values of 83.2%, 77.5%, and 86.6%, respectively. If we pay attention to the false positives (FP) with the three predictive models, there were some coincidences, regardless of the predictive model used. In the logistic regression model, 14 false positives in ABU participants were present in the 23 participants classified as no risk with our cut point of SDUs at the beginning of the prediction. In the classification tree model, of the 31 FPs, 20 were ABU participants labeled as “No Risk,” and of those 31 FPs of the tree, 13 of the 23 FPs of the logistic regression matched. Of those 13, two different classifiers failed, indicating that perhaps the number of weekly SDUs consumption was not real. Finally, paying attention to the FP with the Bayesian network, of the 20 FP, 14 belonged to ABU participants relabeled as “no risk,” again implying that something was happening with the SDUs recognized for these participants. One of the possible reasons for these numerous coincidences may be that people with alcohol use disorders sometimes minimized their consumption and frequency. Therefore, although according to our cut point, they were labeled as “no risk,” their blood test parameters applied to the regression model predicted that they consume alcohol. We checked the medical histories of these participants, and most of them had alcohol-related medical problems in their personal medical history (trauma, heart attack, transient ischemic accident, cerebral vasculopathy, pancreatitis, legal problems…), so we have the hypothesis that they could have minimized the consumption of alcohol.

Only a few studies have attempted the combined use of biomarkers and data science. Some articles combined screening questionaries with some biomarkers, presenting a sensitive tool for detecting alcohol use. The study of [46] combined CAGE with GGT, and they guessed a good tool for detecting alcohol dependence. Another one of [47] combined two different markers (CDT and GGT) to increase diagnostic accuracy for alcohol use disorder. 

Other studies combined biological markers with a questionnaire and clinical parameters for improving the recognition of heavy drinkers [48]. It is similar to our Bayesian network in that one of the selected variables for the best prediction is “educational level,” clinical information that is easy to obtain in a patient’s interview.

A recent study found an association of the elevation of 2-hydroxy-3-methylbutyric with self-reported alcohol intake, and its higher level was associated with the risk of hepatocarcinoma (OR = 2.54; 95%CI = 1.51–4.27) and pancreatic cancer (OR = 1.43; 95% CI 1.03–1.99) [4]. This other study [49] combined two indirect biomarkers (mean corpuscular volume and CDT) with one direct biomarker (Peth) for detection of alcohol ingestion, and they compared the results with a measure of blood alcohol concentration. 

This study [50] validated a new method for the simultaneous determination of four alcohol biomarkers, namely, ethyl glucuronide (EtG), ethyl sulfate, N-acetyltaurine, and 16:0/18:1-phosphatidylethanol (Peth). It was developed and validated using human whole blood. Although the N-acetyltaurine needs further studies for global use, and an adequate cut-off concentration has to be defined, the parallel detection of EtG and Peth in one chromatographic method contributes to a more precise result than detection separately. 

This study serves as a focus point to search for efficient combinations of direct markers, and thus in the future, studying their pharmacokinetics and pharmacodynamics could help to better understand the different patterns of alcohol consumption, as well as being able to prevent the complicated alcohol withdrawal syndrome. Nowadays, these blood determinations are not universalized and are not accessible to most hospitals, so for now, we can use the combinations of indirect markers to improve diagnosis. 

The present study had some strengths, including a sufficient sample size (n = 334) and a clinically strict diagnosis for AUD by experts in the field, from addictive unit care. We checked all drugs [51] people took as chronic treatment and the presence of active viral hepatitis that could affect the biomarkers values (transaminases). We excluded people from blood donors with AUDIT score > 8 for men and >6 for women to ensure that almost every patient from that group was not exposed or minimally exposed to alcohol. Furthermore, we divided the two groups for the predicting model to avoid that bias, depending on the cut point of SDUs. For that reason, in the predicted model, some people from the ABU group received the label “no risk,” but none of the blood donors received the label “risk.”

However, this study had several limitations. First, the retrospective part was based on the clinical history of ABU participants; thus, we consider that the predicted model´s sensitivity and specificity would have been better if we had an objective measure of SDUs, and not only the patient’s version. In the future, we plan to conduct a prospective study, with an alcohol test (breathalyze) of participants, to not need the SDU cut-point. 

Secondly, we did not use the CDT, which is one of the most specific biomarkers [52], but this was because our laboratory does not use this biomarker routinely, and we wanted a tool that could be used in any consultation at primary care or hospitalized participants with the standard basic blood test. 

Thus far, as there is no ideal validated biomarker, which is essential to explore new combinations of biomarkers [10] and discover new ways to detect alcohol use with inexpensive and accessible tools, using data science combined with medical knowledge. We are currently working on a project, creating an app for smartphones or computers, where you can fill in the biomarker value in each box to obtain the result of the predicted model (Bayesian network), so it will be easy to use and you will be able to do it at the patient’s bedside. 

A future study will be to validate the predictive model by a computer or smartphone app, where it will be possible to write the biomarker value and receive from the application the predicted label. Secondly, another project will be to create a system for detecting when a patient underestimates the alcohol quantity; for example, 14 participants were labeled as no risk, and the predicted model classified them as at risk. Is the predictive model making mistakes, or did the patient not say the correct alcohol quantity consumed? In addition, 10 of them were labelled as at risk for the three predicted models, so something about it has to be resolved in the future. 

## 5. Conclusions

By combining several biochemical markers, clinical history (study level), and machine learning, we can enhance the detection of AUD, helping to prevent future complications from alcohol use. Our best predictive model to predict risky alcohol consumption, with an accuracy of 87.5%, using a Bayesian network, selected the best combination of nine biomarkers (basophils, creatinine, alkaline phosphatase, gamma-glutamyl transferase, mean corpuscular haemoglobin, hematocrit, red blood cells distribution width, lactate dehydrogenase, and urea) and the study’s level, being able to predict if the biomarkers alterations of our patients could be secondary to alcohol consumption. It had a sensitivity of 88.3% and a specificity of 86.6%. The selected variables were easy to obtain, even in emergency services, so if the model was validated, it could be an accessible tool for several doctors in many departments (emergencies, traumatology, psychiatry, internal medicine, general practice doctor), advocating for an early diagnosis of AUD and anticipating the possible problems derived from its consumption. 

## Figures and Tables

**Table 1:** Coefficients calculated in logistic regression, for all the variables used, together with the confidence interval and its *p*-value. Statistically significant ones are marked with *.

Variable	Coefficient	Confidence Interval (95%)		*p* Value
Constant	0.826	0.084	1.569	0.029
Study level	−0.424	−0.973	0.125	0.130
Age	−0.191	−0.746	0.364	0.499
Albumin	0.497	−1.215	2.209	0.570
Uric acid	−0.057	−0.635	0.521	0.846
Basophils	−1.907	−4.863	1.050	0.206
Basophils %	1.898	−0.766	4.562	0.163
Total bilirubin	0.283	−0.921	1.487	0.645
Calcium	0.284	−0.493	1.061	0.474
MCHC ^1^	−8.259	−15.893	−0.625	0.034 *
Chlorine	1.029	0.320	1.737	0.004 *
Cholesterol	0.826	−0.614	2.267	0.261
Creatinine	−0.608	−1.322	0.106	0.095
Eosinophils	−12.068	−23.030	−1.106	0.031 *
Eosinophils %	11.639	−3.425	26.702	0.130
Red blood cells	−2.775	−10.355	4.804	0.473
Alkaline phosphatase	1.125	0.244	2.006	0.012 *
Ferritin	1.872	0.268	3.477	0.022 *
Gamma glutamyl transferase	−0.044	−2.222	2.134	0.968
Globulins	0.533	−1.112	2.177	0.526
Glucose	−0.483	−1.082	0.115	0.114
Aspartate aminotransferase	0.423	−1.778	2.624	0.706
Alanine aminotransferase	0.503	−0.554	1.559	0.351
Haemoglobin	−8.424	−21.414	4.566	0.204
Mean corpuscular haemoglobin	23.651	7.540	39.762	0.004 *
Hematocrit	11.208	−2.673	25.089	0.114
HDL- cholesterol	−1.033	−2.066	0.001	0.050
Red blood cells distribution width	0.818	0.158	1.477	0.015 *
Platelet distribution width	0.491	−0.176	1.158	0.149
Potassium	0.636	0.014	1.259	0.045 *
Lactate dehydrogenase	0.412	−0.193	1.016	0.182
LDL-cholesterol	0.310	−0.869	1.489	0.607
White blood cells	205.737	15.672	395.801	0.034 *
Lymphocytes	−59.007	−113.951	−4.063	0.035 *
% Lymphocytes	50.220	−20.111	120.550	0.162
% Large Unstained Cells	17.875	−9.300	45.049	0.197
Large Unstained Cells	−20.052	−42.138	2.034	0.075
Monocytes	−14.328	−26.135	−2.521	0.017 *
% Monocytes	12.3017	−2.676	27.280	0.107
Myeloperoxidase index	−0.180	−0.757	0.397	0.541
Neutrophils	−185.847	−358.363	−13.331	0.035 *
% Neutrophils	60.0477	−22.608	142.704	0.154
Phosphorus	−0.422	−1.032	0.188	0.175
Platelets	−0.114	−0.790	0.563	0.742
Total Proteins	−1.113	−2.745	0.519	0.181
Triglycerides	−0.603	−1.554	0.348	0.214
Transferrin	0.143	−0.582	0.869	0.698
Urea	−1.238	−2.050	−0.426	0.003 *
Mean Corpuscular Volume	−22.442	−36.694	−8.190	0.002 *
Mean Platelet Volume	−0.463	−1.174	0.249	0.202
Sodium	−1.553	−2.430	−0.677	0.001 *
SEX_woman	−0.278	−1.021	0.464	0.462

^1^ red cell mean corpuscular hemoglobin concentration. *: Statistically significant ones.

**Table 2:** Coefficients calculated for logistic regression model for ‘risk’ with automatic selection (IWSSr), for the variables used, together with the confidence interval and its *p* value.

Variable	Coefficient	Confidence Interval (95%)		*p* Value
Constant	0.7079	0.263	1.153	0.002
Mean corpuscular haemoglobin	1.3679	0.944	1.791	0
Gamma glutamyl transferase	2.78	1.112	4.448	0.001
Red blood cells distribution width	1.0657	0.663	1.469	0
Creatinine	−0.7371	−1.089	−0.385	0
Total bilirubin	0.4942	−0.173	1.161	0.146
Mean Platelet Volume	−0.1804	−0.479	0.118	0.236
Large Unstained Cells	0.4084	−0.938	1.755	0.552
HDL-cholesterol	−0.676	−1.093	−0.259	0.002

**Table 3:** Probability table for risk and the discretized variables in the Bayesian network.

Variable	Value	Risk	No risk
Gamma glutamyl transferase	≤46.5	0.572	0.929
	>46.5	0.428	0.071
Mean corpuscular haemoglobin	<31.3	0.25	0.8
	≥31.3	0.75	0.2
Educational level	1	0.020	0.003
	2	0.202	0.033
	3	0.526	0.282
	4	0.122	0.245
	5	0.082	0.312
	6	0.048	0.124
Basophils	≤0.01	0.009	0.107
	(0.01–0.015]	0.077	0.027
	(0.015–0.02]	0.003	0.216
	>0.02	0.911	0.649
Creatinine	≤1.025	0.871	0.598
	>1.025	0.129	0.402
Alkaline phosphatase	≤34	0.003	0.131
	(34–84.5]	0.771	0.810
	>54.5	0.226	0.058
Hematocrit	≤47.09	0.618	0.850
	>47.09	0.382	0.150
Red blood cells distribution width	≤13.05	0.141	0.469
	>13.05	0.859	0.531
Lactate Dehydrogenase	≤256.5	0.836	0.985
	>256.5	0.164	0.015
Urea	≤23.5	0.415	0.070
	(23.5–40.5]	0.519	0.566
	>40.5	0.066	0.364

Please note ‘(‘ stands for ‘open range value’ and ‘]’ stands for ‘closed range value’. For instance, (0.01 means any value greater than 0.1 but not equal; and 0.015] means exactly 0.015.

## Data Availability

Here, you can access to the dataset for the predictive model. It is the public repository link from the official account of Intelligent Systems and Data Mining Group (Grupo Sistemas Inteligentes y Minería de datos (SIMD)) from the University of Castilla La Mancha (Universidad de Castilla La Mancha (UCLM)). https://github.com/UCLM-SIMD/alcohol_risk_prediction. (last access and update 25 October 2021).

## Supplementary Materials

The following supporting information can be downloaded at: https://www.mdpi.com/article/10.3390/jcm11072061/s1. Table S1. Variables´s definition used in the predictive model. Figure S1. Excerpt from the notebook generated with the predictive analysis with Scikit-learn. Figure S2. Use of the Weka application for the creation of predictive models. Table S2. Correlation of different laboratory parameters with the binomial variable risk/no risk (alcohol consume). Figure S3. Correlation of lab parameters with alcohol consumption (in Standard Drink Units (SDU). Figure S4. Rate of participants with alcohol consumption risk. Example S1. Example of manual use of the logistic regression model to make a prediction. Example S2. Example of manual use for bayesian network for prediction.
