# Supplementary material for: Common Laboratory Parameters Are Useful for Screening for Alcohol Use Disorder: Designing a Predictive Model Using Machine Learning
Source: J Clin Med. 2022 Apr 6;11(7):2061. doi: 10.3390/jcm11072061 (PMC8999878; doi:10.3390/jcm11072061)
Supplement: Supplementary file 1 [file jcm-11-02061-s001.zip › jcm-1590356-supplementary.pdf]

SUPPLEMENTARY INFORMATION

COMMON LABORATORY PARAMETERS ARE USEFUL FOR SCREENING FOR ALCOHOL USE DISORDER. DESIGNING A PREDICTIVE MODEL USING MACHINE LEARNING.

Juana Pinar-Sanchez [1], Pablo Bermejo López [2,*], Julián Solís García del Pozo [3,*], Jose Redondo-Ruiz [4,*], Laura Navarro Casado [5], Fernando Andres-Pretel [6], María Luisa Celorrio Bustillo [7], Mercedes Esparcia Moreno [8], Santiago García Ruiz [9], Jose Javier Solera Santos [10] and Beatriz Navarro Bravo [11,*].

[1] Department of Internal Medicine. Jose Maria Morales Meseguer University General Hospital. 30008. Murcia. Spain.

[2] Associate Professor. Universidad de Castilla-La Mancha, Computer Science Department. 02071. Albacete. Spain.

[3] Department of Internal Medicine. Unit of Infectious Diseases. University General Hospital of Albacete. 02006. Albacete. Spain.

[4] Department of dermatology, stomatology, radiology and physical medicine. Special care dentistry unit and gerodontology. Jose Maria Morales Meseguer University General Hospital. Faculty of Medicine. University of Murcia. 30008. Murcia. Spain

[5] Department of Biochemistry. University General Hospital of Albacete. 02006. Albacete. Spain.

[6] Clinical Research Support Unit. National Paraplegics Hospital of Toledo Foundation. 45004. Toledo. Spain.

[7] Department of Mental Health: Addictive Conducts Unit Care in Albacete. General Practitioner and Master in Drug Addiction and Alcoholism. 02005. Albacete. Spain.

[8] Department of Mental Health: Addictive Conducts Unit Care in Albacete. Psychologist. Master in Drug Addiction and Alcoholism. 02005. Albacete. Spain.

[9] General Practitioner, blood donation center from Albacete and Cuenca. Department of Hematology. University General Hospital of Albacete. 02006. Albacete. Spain.

[10] Department of Internal Medicine. University General Hospital of Albacete. 02006. Albacete. Spain.

[11] Department of Psychology. Professor in Faculty of Medicine. Universidad de Castilla-La Mancha. 02008. Albacete. Spain.

* Corresponding authors:

beatriz.navarro@uclm.es (B.N.B.); Tel: + (34) 967 599 200, 96150 (conserjería) / ext. 2902 (decanato); julianeloysolis@gmail.com (J.S.G.d.P.); Tel: 967 597 214 (Internal Medicine/Unit of Infectious Diseases). University General Hospital of Albacete).

Supplementary information: Table S1. Variables´s definition used in the predictive model.

**Variables´s name**	**Definition**	**Units**
ID	Code number	
CASES	Type of participant	
STUDY_LEVEL	Study_level	
MARITAL_STATUS	Marital_status	
SEX	Sex	
AGE	Age	years
ALB	Albumin	g/dL
AMI	Amylase	g/dL
APTT	Activated Partial Thromboplastin Time	seconds
UA	Uric acid	mg/dL
BAS	Basophils	x10^3mcl
BAS_PERCENT	Basophils_Percent	%
DBIL	Direct Bilirubin	mg/dL
IBIL	Indirect Bilirubin	mg/dL
TBIL	Total Bilirubin	mg/dL
CA	Calcium	mg/dL
MCHC	Mean Corpuscular Hemoglobin Concentration	g/dL
CK	Creatine Kinase	ng/mL
CL	Chlorine	mmol/L
COAG_	Coagulation	g/dL
CHOL	Cholesterol	mg/dL
CREA	Creatinine	mg/dL
EOS	Eosinophils	x10^3mcl
EOS_PERCENT	Eosinophils_Percent	%
RBC	Red Blood Cells (RBC)	x10^6mcl
ALP	Alkaline Phosphatase	U/L
FERRIT	Ferritin	ng/mL
Fibri	Fibrinogen C	mg/dL
GGT	Gamma Glutamyl Transferase	U/L
GLOB	Globulins	g/dL
GLU	Glucose	mg/dL
AST	Aspartate aminotransferase	U/L
ALT	Alanine aminotransferase	U/L
HB	Hemoglobin	g/dL
MCH	Mean Corpuscular Hemoglobin	pg
HCT	Hematocrit	%
HDL	High-Density Lipoprotein Cholesterol	mg/dL

RDW	Red Blood Cells Dispersion Index	%
PDW	Platelet Dispersion Index	%
INR_	International Normalized Ratio	
K	Potassium	mmol/L
LDH	Lactate dehydrogenase	U/L
LDL	Low-density lipoprotein cholesterol	mg/dL
WBC	White blood cells (WBC)	x10^3mcl
LYM	Lymphocites	x10^3mcl
LYM_PERCENT	Lymphocites_Percent	%
LUC_PERCENT	Large Unstained Cells_Percent	%
LUC_	Large Unstained Cells	x10^3mcl
MONOS	Monocytes	x10^3mcl
MONOS_PERCENT	Monocytes_Percent	%
MPO	Myeloperoxidase index	
NEUTR	Neutrophils	x10^3mcl
NEUTR_PERCENT	Neutrophils_Percent	%
P	Phosphorus	mg/dL
CRP	C-Reactive Protein	mg/dL
PLAT	Platelets	x10^3mcl
TP	Total Proteins	g/dL
TG	Trygliceridesq	mg/dL
Tf	Transferrin	mg/dL
UREA	Urea	mg/dL
MCV	Mean Corpuscular Volume	fL
MPV	Mean Platelet Volume	fL
SODIUM	Na (Sodium)	mmol/L
WEEKLY_SDU	Weekly_Standard Drink Units	1SDU=10g alcohol

Supplementary information: Figure S1. Excerpt from the notebook generated with the predictive analysis with Scikit-learn.

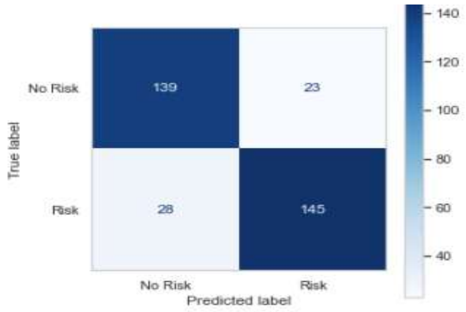


```
params = last_result_IWSSr.params
params_iwssr =last_result_IWSSr.params
conf = last_result_IWSSr.conf_int()
conf['OR'] = params
cols= ['2.5%', '97.5%', 'OR']
conf.columns =cols
cols = cols[-1:] + cols[:-1]
conf = conf[cols]
df_iwssr_params=np.exp(conf)
df_iwssr_params['Coefs']=params_iwssr
df_iwssr_params=df_params[['Coefs','OR','2.5%','97.5%']]
display(df_iwssr_params)
```

	Coefs	OR	2.5%	97.5%
const	0.707872	2.029667	1.300970	3.166521
MCH	1.367883	3.927028	2.571032	5.998193
GGT	2.780029	16.119496	3.041074	85.442877
RDW	1.065720	2.902929	1.940129	4.343525
CREA	-0.737093	0.478503	0.336430	0.680574
TBIL	0.494245	1.639260	0.841488	3.193356
MPV	-0.180432	0.834910	0.619321	1.125546
LUC_	0.408417	1.504434	0.391252	5.784825
HDL	-0.676012	0.508641	0.335043	0.772187

Supplementary information: Figure S2. Use of the Weka application for the creation of predictive models.

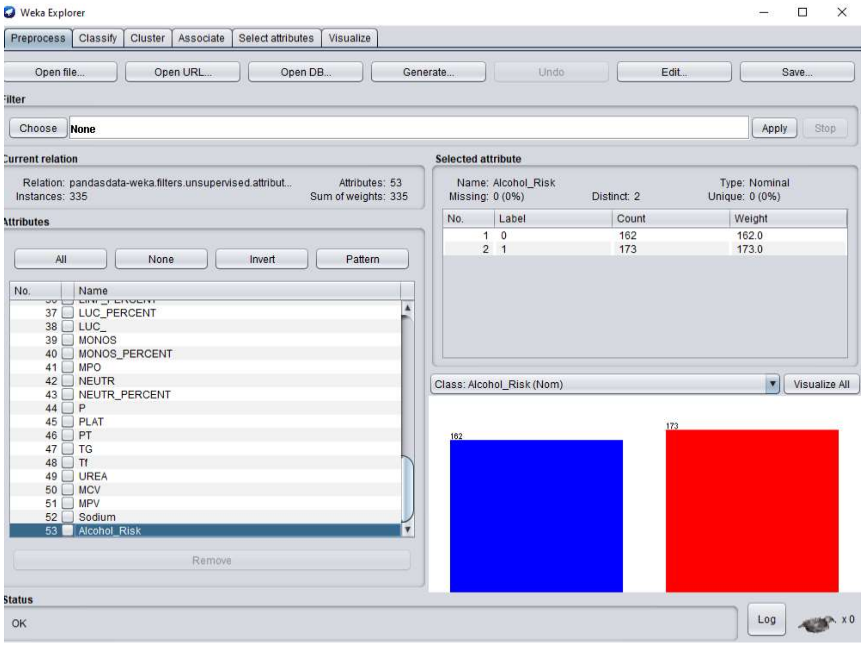

Supplementary information: Table S2. Correlation of different laboratory parameters with the binomial variable risk/no risk (alcohol consume).

S. 2.1. Men and women.

Risk drinker / Variables	No (CI 95%)	Yes (CI 95%)	p-value
Albumin	4.745 [4.699,4.791]	4.465 [4.363,4.568]	2.94E-05*
Uric acid	5.336 [5.119,5.553]	5.400 [5.112,5.688]	9.77E-01
Basophils	0.038 [0.034,0.042]	0.042 [0.039,0.046]	1.68E-04*
% Basophils	0.454 [0.415,0.493]	0.525 [0.481,0.568]	1.26E-01
Total Bilirrubin	0.449 [0.402,0.496]	0.660 [0.521,0.798]	7.21E-02
Calcium	9.374 [9.317,9.431]	9.292 [9.173,9.411]	3.15E-01
Medium corpuscular hemoglobin concentration	33.170 [32.980,33.360]	33.363 [33.175,33.550]	1.56E-01
Chlorine	100.747 [100.373,101.121]	101.235 [100.552,101.918]	8.72E-02
Total Cholesterol	192.468 [187.586,197.349]	206.144 [197.494,214.794]	1.70E-02*
Creatinine	0.973 [0.941,1.005]	0.840 [0.811,0.868]	1.56E-09*
Eosinophils	0.228 [0.209,0.247]	0.222 [0.195,0.249]	7.34E-02
% Eosinophils	2.804 [2.580,3.029]	2.736 [2.425,3.047]	1.11E-01
Red blood cells	4.851 [4.762,4.940]	4.697 [4.608,4.786]	4.29E-03*
Alkaline Phosphatase	55.873 [52.025,59.721]	82.144 [75.070,89.219]	2.79E-09*
Ferritin	74.014 [60.099,87.928]	306.255 [232.433,380.077]	3.82E-17*
Gamma glutamyl transferase	29.255 [22.662,35.847]	149.830 [96.124,203.535]	1.04E-20*
Globulins	2.438 [2.387,2.490]	2.590 [2.476,2.704]	2.84E-02*
Glucose	85.786 [81.231,90.342]	96.970 [91.355,102.585]	1.41E-06*
Aspartate aminotransferase (AST)	21.194 [19.968,22.420]	46.444 [38.021,54.867]	1.17E-10*
Alanine aminotransferase (ALT)	21.924 [20.225,23.623]	40.261 [34.639,45.883]	4.74E-09*

Total Hemoglobin	14.448 [14.172,14.725]	15.068 [14.810,15.326]	1.17E-04*
Mean Corpuscular Hemoglobin	29.840 [29.520,30.160]	32.207 [31.865,32.549]	2.66E-22*
Hematocrit	43.507 [42.746,44.269]	45.169 [44.419,45.919]	2.75E-04*
HDL (High Density Lipoprotein) - cholesterol	57.208 [53.616,60.800]	60.234 [55.080,65.388]	7.76E-01
Red Blood Cells distribution width	13.419 [13.216,13.621]	14.284 [14.031,14.536]	1.31E-08*
Platelet distribution width	51.253 [50.186,52.321]	51.262 [50.009,52.516]	8.99E-01
Potassium	4.323 [4.267,4.380]	4.481 [4.403,4.559]	1.43E-03*
Lactate dehydrogenase	180.176 [174.705,185.646]	217.388 [201.024,233.753]	1.36E-02*
LDL (Light Density Lipoprotein)-Cholesterol	104.357 [97.827,110.887]	121.852 [114.450,129.253]	1.32E-03*
White blood cells	8.308 [7.994,8.623]	8.509 [8.017,9.000]	5.92E-01
Lymphocytes	2.576 [2.474,2.679]	2.336 [2.203,2.468]	1.06E-03*
% Lymphocytes	31.705 [30.594,32.816]	29.080 [27.639,30.521]	9.05E-03*
% Large Unstained Cells	2.058 [1.953,2.163]	2.472 [1.788,3.155]	7.56E-01
Large Unstained Cells	0.167 [0.159,0.175]	0.200 [0.139,0.261]	3.40E-01
Monocytes	0.513 [0.492,0.534]	0.513 [0.485,0.541]	6.23E-01
Monocytes%	6.298 [6.074,6.522]	6.361 [6.063,6.658]	6.16E-01
Myeloperoxidase	-1.264 [-2.089,-0.439]	0.234 [-0.622,1.089]	8.78E-03*
Sodium	142.474 [142.166,142.782]	140.820 [140.235,141.404]	1.89E-07*
Neutrophils	4.787 [4.526,5.049]	5.197 [4.738,5.655]	9.70E-01
% Neutrophils	56.672 [55.436,57.908]	58.821 [57.055,60.586]	5.83E-02
Phosphor	3.501 [3.420,3.581]	3.225 [3.098,3.353]	1.48E-03*
Platelets	238.913 [228.327,249.499]	244.576 [232.566,256.587]	2.55E-01
Total Protein	7.184 [7.125,7.244]	7.049 [6.947,7.151]	1.17E-02*

Tryglicerides	162.604 [146.138,179.070]	136.063 [115.439,156.688]	4.87E-03*
Transferrin	303.800 [296.494,311.106]	266.511 [255.825,277.197]	2.79E-08*
Urea	36.196 [34.787,37.606]	26.719 [25.098,28.339]	2.35E-17*
Mean Corpuscular Volume	89.979 [89.092,90.866]	96.551 [95.596,97.505]	1.31E-20*
Mean Platelet Volume	9.488 [9.315,9.660]	9.454 [9.266,9.643]	5.63E-01

S. 2.2. Men.

**Risk drinker**			
**Variables**	**No (CI 95%)**	**Yes (CI 95%)**	**p-value**
Albumin	4.759 [4.709,4.810]	4.523 [4.415,4.631]	8.87E-04*
Uric acid	5.770 [5.541,5.999]	5.659 [5.337,5.981]	3.00E-01
Basophils	0.034 [0.031,0.038]	0.044 [0.040,0.048]	5.53E-06*
% Basophils	0.428 [0.385,0.471]	0.528 [0.478,0.577]	4.76E-02*
Total Bilirrubin	0.498 [0.441,0.554]	0.677 [0.510,0.843]	7.53E-01
Calcium	9.384 [9.318,9.449]	9.306 [9.169,9.442]	3.46E-01
Medium corpuscular hemoglobin concentration	33.407 [33.192,33.622]	33.456 [33.258,33.654]	9.71E-01
Chlorine	100.609 [100.136,101.082]	101.208 [100.471,101.944]	7.10E-02
Total Cholesterol	193.835 [187.769,199.901]	206.912 [197.483,216.341]	5.23E-02
Creatinine	1.032 [0.998,1.067]	0.863 [0.833,0.892]	1.09E-12*
Eosinophils	0.236 [0.212,0.260]	0.223 [0.194,0.251]	8.32E-02
% Eosinophils	2.971 [2.705,3.238]	2.686 [2.375,2.996]	2.19E-02*
Red blood cells	4.965 [4.853,5.076]	4.825 [4.730,4.919]	5.47E-03*
Alkaline Phosphatase	61.457 [57.204,65.710]	81.659 [74.442,88.875]	3.96E-05*
Ferritin	81.495 [64.582,98.408]	323.063 [244.884,401.242]	1.24E-17*

Gamma glutamyl transpeptidase	31.027 [23.366,38.687]	152.879 [87.705,218.052]	7.88E-14*
Globulins	2.390 [2.332,2.448]	2.561 [2.437,2.685]	1.59E-02*
Glucose	88.816 [82.641,94.991]	96.652 [90.979,102.324]	1.05E-03*
Aspartate aminotransferase (AST)	22.257 [21.029,23.484]	45.299 [36.724,53.873]	1.20E-05*
Alanine aminotransferase (ALT)	24.286 [22.269,26.303]	40.826 [34.194,47.457]	6.16E-05*
Total Hemoglobin	14.917 [14.594,15.241]	15.457 [15.207,15.707]	9.30E-04*
Mean Corpuscular Hemoglobin	30.120 [29.787,30.453]	32.169 [31.837,32.500]	5.46E-16*
Hematocrit	44.616 [43.695,45.537]	46.237 [45.462,47.011]	4.70E-04*
HDL (High Density Lipoprotein) - Cholesterol	53.250 [48.969,57.531]	58.200 [52.169,64.231]	8.13E-01
Red Blood Cells distribution width	13.243 [13.024,13.461]	14.146 [13.898,14.394]	6.35E-09*
Platelet distribution width	51.738 [50.436,53.041]	51.604 [50.138,53.070]	7.03E-01
Potassium	4.328 [4.262,4.395]	4.487 [4.399,4.576]	5.26E-03*
Lactate dehydrogenase	181.170 [174.699,187.641]	214.773 [196.336,233.211]	1.61E-01
LDL (Light Density Lipoprotein)- Cholesterol	104.979 [96.390,113.567]	123.575 [115.626,131.523]	2.98E-03*
White blood cells	8.021 [7.668,8.373]	8.559 [8.104,9.014]	1.93E-01
Lymphocytes	2.503 [2.382,2.623]	2.349 [2.210,2.488]	7.11E-02
% Lymphocytes	31.723 [30.465,32.982]	28.628 [27.017,30.239]	4.96E-03*
% Large Unstained Cells	2.122 [1.994,2.250]	2.131 [1.964,2.298]	6.41E-01
Large Unstained Cells	0.167 [0.156,0.177]	0.174 [0.161,0.187]	8.37E-01
Monocytes	0.524 [0.498,0.550]	0.534 [0.503,0.565]	8.03E-01
Monocytes%	6.627 [6.353,6.901]	6.460 [6.150,6.770]	3.61E-01
Myeloperoxidase	-1.186 [-2.184,-0.188]	-0.006 [-0.953,0.941]	6.54E-02

Sodium	142.964 [142.616,143.311]	141.085 [140.452,141.718]	1.05E-08*
Neutrophils	4.559 [4.280,4.837]	5.236 [4.834,5.638]	5.74E-02
% Neutrophils	56.121 [54.730,57.511]	59.563 [57.789,61.338]	7.46E-03*
Phosphor	3.522 [3.426,3.619]	3.225 [3.080,3.371]	3.36E-03*
Platelets	224.739 [213.499,235.980]	240.493 [227.210,253.775]	2.67E-02*
Total Protein	7.151 [7.080,7.222]	7.073 [6.955,7.192]	1.77E-01
Tryglicerides	179.807 [159.881,199.734]	141.892 [116.809,166.976]	2.38E-04*
Transferrin	296.699 [288.220,305.178]	258.806 [247.280,270.331]	1.31E-07*
Urea	38.000 [36.397,39.603]	27.425 [25.547,29.303]	4.83E-16*
Mean Corpuscular Volume	90.193 [89.259,91.127]	96.190 [95.214,97.165]	2.14E-15*
Mean Platelet Volume	9.395 [9.194,9.595]	9.392 [9.185,9.599]	7.84E-01

S. 2.3. Women.

**Risk drinker** / **Variables**	**No (CI 95%)**	**Yes (CI 95%)**	**p-value**
Albumin	4.693 [4.592,4.793]	4.213 [3.941,4.485]	6.70E-04*
Uric acid	4.144 [3.847,4.441]	4.364 [3.881,4.847]	4.01E-01
Basophils	0.046 [0.037,0.054]	0.037 [0.031,0.043]	9.36E-01
% Basophils	0.507 [0.427,0.587]	0.514 [0.422,0.605]	9.58E-01
Total Bilirrubin	0.306 [0.245,0.367]	0.596 [0.370,0.822]	3.18E-03*
Calcium	9.333 [9.215,9.452]	9.235 [8.985,9.485]	4.87E-01
Medium corpuscular hemoglobin concentration	32.558 [32.205,32.911]	33.017 [32.522,33.512]	2.92E-02*
Chlorine	101.116 [100.526,101.706]	101.346 [99.494,103.199]	8.43E-01

Total Cholesterol	189.977 [181.736,198.218]	202.739 [179.612,225.866]	3.16E-01
Creatinine	0.810 [0.761,0.859]	0.752 [0.675,0.829]	1.45E-02*
Eosinophils	0.201 [0.170,0.232]	0.219 [0.149,0.289]	3.67E-01
% Eosinophils	2.291 [1.926,2.656]	2.922 [1.980,3.865]	4.94E-01
Red blood cells	4.541 [4.443,4.639]	4.222 [4.072,4.372]	6.68E-04*
Alkaline Phosphatase	42.365 [35.622,49.108]	84.316 [60.987,107.644]	2.62E-04*
Ferritin	53.545 [29.299,77.792]	248.522 [49.379,447.665]	2.05E-02*
Gamma glutamyl transpeptidase	25.091 [11.520,38.661]	138.179 [60.733,215.625]	1.78E-07*
Globulins	2.553 [2.451,2.654]	2.723 [2.404,3.042]	6.27E-01
Glucose	78.273 [75.078,81.468]	98.206 [81.105,115.307]	2.59E-04*
Aspartate aminotransferase (AST)	17.326 [16.079,18.574]	50.829 [25.762,75.895]	3.94E-07*
Alanine aminotransferase (ALT)	15.978 [13.399,18.556]	38.138 [27.743,48.533]	7.31E-06*
Total Hemoglobin	13.200 [12.868,13.532]	13.622 [13.024,14.221]	8.89E-02
Mean Corpuscular Hemoglobin	29.133 [28.382,29.885]	32.350 [31.262,33.438]	1.01E-06*
Hematocrit	40.538 [39.613,41.463]	41.194 [39.688,42.701]	2.72E-01
HDL (High Density Lipoprotein) - Cholesterol	65.435 [59.556,71.313]	68.952 [60.542,77.362]	8.88E-01
Red Blood Cells distribution width	13.873 [13.425,14.322]	14.794 [14.033,15.556]	6.59E-02
Platelet distribution width	50.027 [48.127,51.926]	49.992 [47.620,52.363]	9.70E-01
Potassium	4.314 [4.200,4.428]	4.454 [4.279,4.628]	1.87E-01
Lactate dehydrogenase	174.341 [165.876,182.807]	227.958 [190.073,265.844]	4.59E-03*
LDL (Light Density Lipoprotein)-Cholesterol	103.955 [93.377,114.532]	114.714 [94.317,135.112]	4.81E-01
White blood cells	9.051 [8.401,9.700]	8.324 [6.670,9.977]	5.52E-03*
Lymphocytes	2.754 [2.557,2.951]	2.286 [1.916,2.655]	1.68E-03*

% Lymphocytes	31.507 [29.101,33.912]	30.764 [27.452,34.076]	9.05E-01
% Large Unstained Cells	1.896 [1.712,2.079]	3.742 [0.485,6.998]	1.80E-01
Large Unstained Cells	0.168 [0.152,0.184]	0.296 [0.005,0.587]	6.38E-02
Monocytes	0.490 [0.455,0.524]	0.436 [0.379,0.493]	1.80E-01
Monocytes%	5.493 [5.219,5.768]	5.992 [5.164,6.819]	2.25E-02*
Myeloperoxidase	-1.387 [-2.926,0.153]	1.125 [-0.911,3.161]	4.44E-02*
Sodium	141.209 [140.713,141.705]	139.778 [138.300,141.255]	1.60E-01
Neutrophils	5.394 [4.805,5.982]	5.051 [3.423,6.679]	2.28E-03*
% Neutrophils	58.296 [55.660,60.931]	56.056 [50.850,61.261]	3.25E-01
Phosphor	3.436 [3.284,3.588]	3.225 [2.931,3.519]	2.10E-01
Platelets	272.778 [250.956,294.599]	259.778 [231.230,288.326]	4.11E-01
Total Protein	7.245 [7.138,7.352]	6.940 [6.747,7.133]	7.08E-03*
Tryglicerides	122.386 [95.653,149.120]	111.292 [92.591,129.992]	9.49E-01
Transferrin	320.610 [306.965,334.255]	294.250 [269.805,318.695]	1.14E-01
Urea	31.795 [29.249,34.342]	23.848 [20.867,26.830]	1.26E-04*
Mean Corpuscular Volume	89.482 [87.329,91.636]	97.894 [95.151,100.638]	3.77E-06*
Mean Platelet Volume	9.740 [9.397,10.083]	9.686 [9.230,10.142]	7.00E-01

Supplementary information: Figure S3. Correlation of lab parameters with alcohol consumption (in Standard Drink Units (SDU).

S 3.1 Men and women

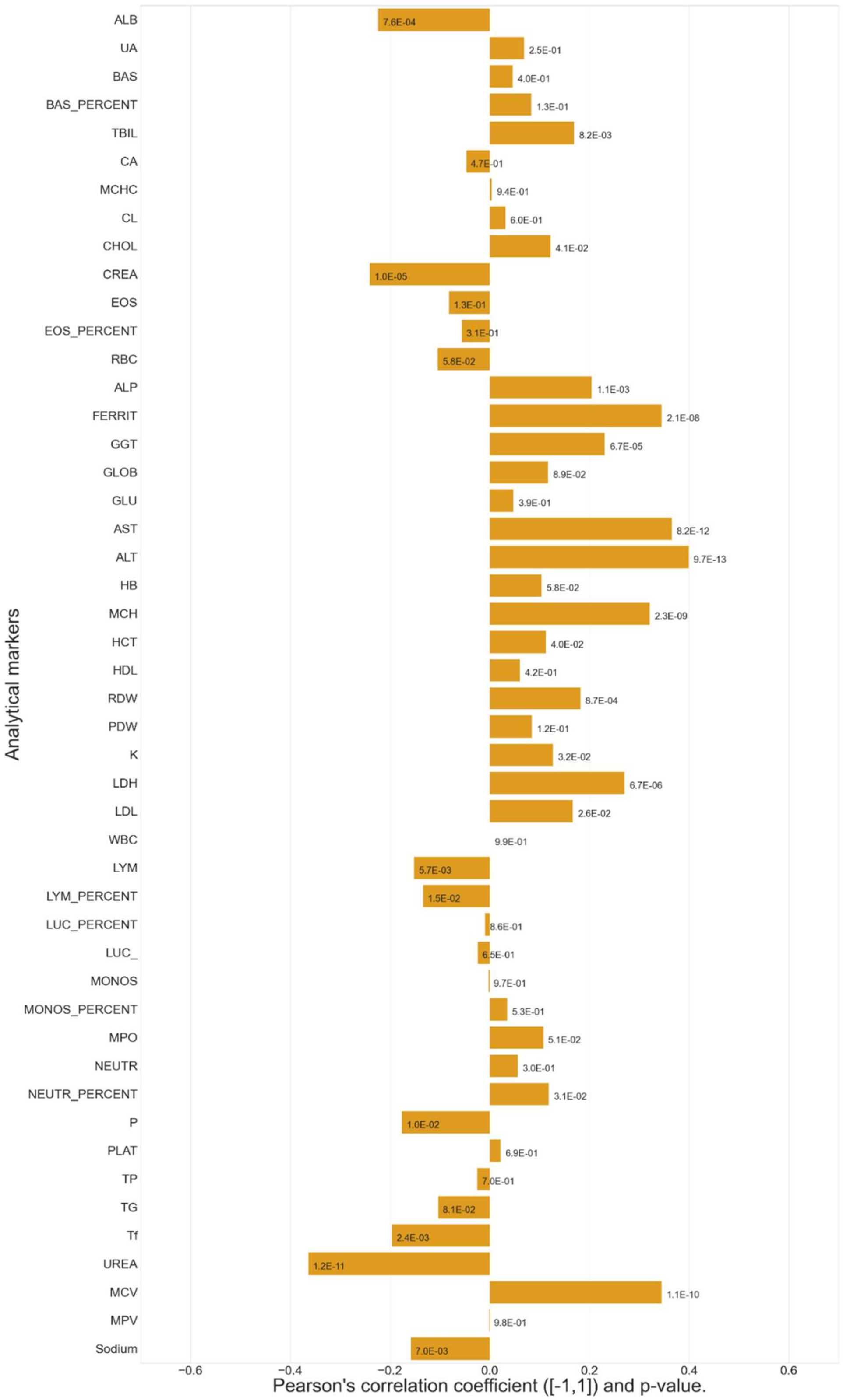

S 3.2. Men

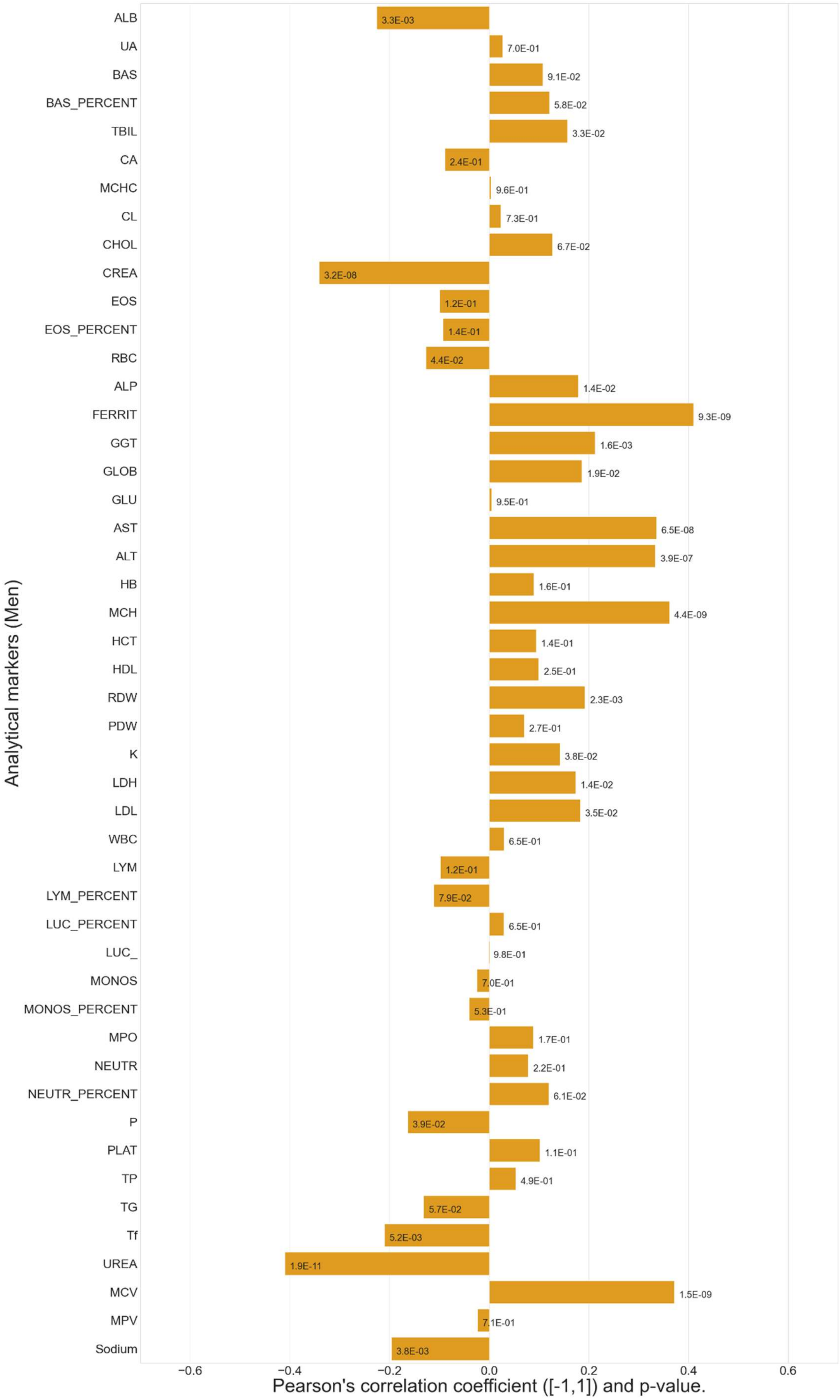
ALB
UA
BAS
BAS_PERCENT
TBIL
CA
MCHC
CL
CHOL
CREA
EOS
EOS_PERCENT
RBC
ALP
FERRIT
GGT
GLOB
GLU
AST
ALT
HB
MCH
HCT
HDL
RDW
PDW
K
LDH
LDL
WBC
LYM
LYM_PERCENT
LUC_PERCENT
LUC_
MONOS
MONOS_PERCENT
MPO
NEUTR
NEUTR_PERCENT
P
PLAT
TP
TG
Tf
UREA
MCV
MPV
Sodium
3.3E-03
7.0E-01
9.1E-02
5.8E-02
3.3E-02
2.4E-01
9.6E-01
7.3E-01
6.7E-02
3.2E-08
1.2E-01
1.4E-01
4.4E-02
1.4E-02
9.3E-09
1.6E-03
1.9E-02
9.5E-01
6.5E-08
3.9E-07
1.6E-01
4.4E-09
1.4E-01
2.5E-01
2.3E-03
2.7E-01
3.8E-02
1.4E-02
3.5E-02
6.5E-01
1.2E-01
7.9E-02
6.5E-01
9.8E-01
7.0E-01
5.3E-01
1.7E-01
2.2E-01
6.1E-02
3.9E-02
1.1E-01
4.9E-01
5.7E-02
5.2E-03
1.9E-11
1.5E-09
7.1E-01
3.8E-03
Analytical markers (Men)
−0.6
−0.4
−0.2
0.0
0.2
0.4
0.6
Pearson's correlation coefficient ([-1,1]) and p-value.

S 3.3. Women.

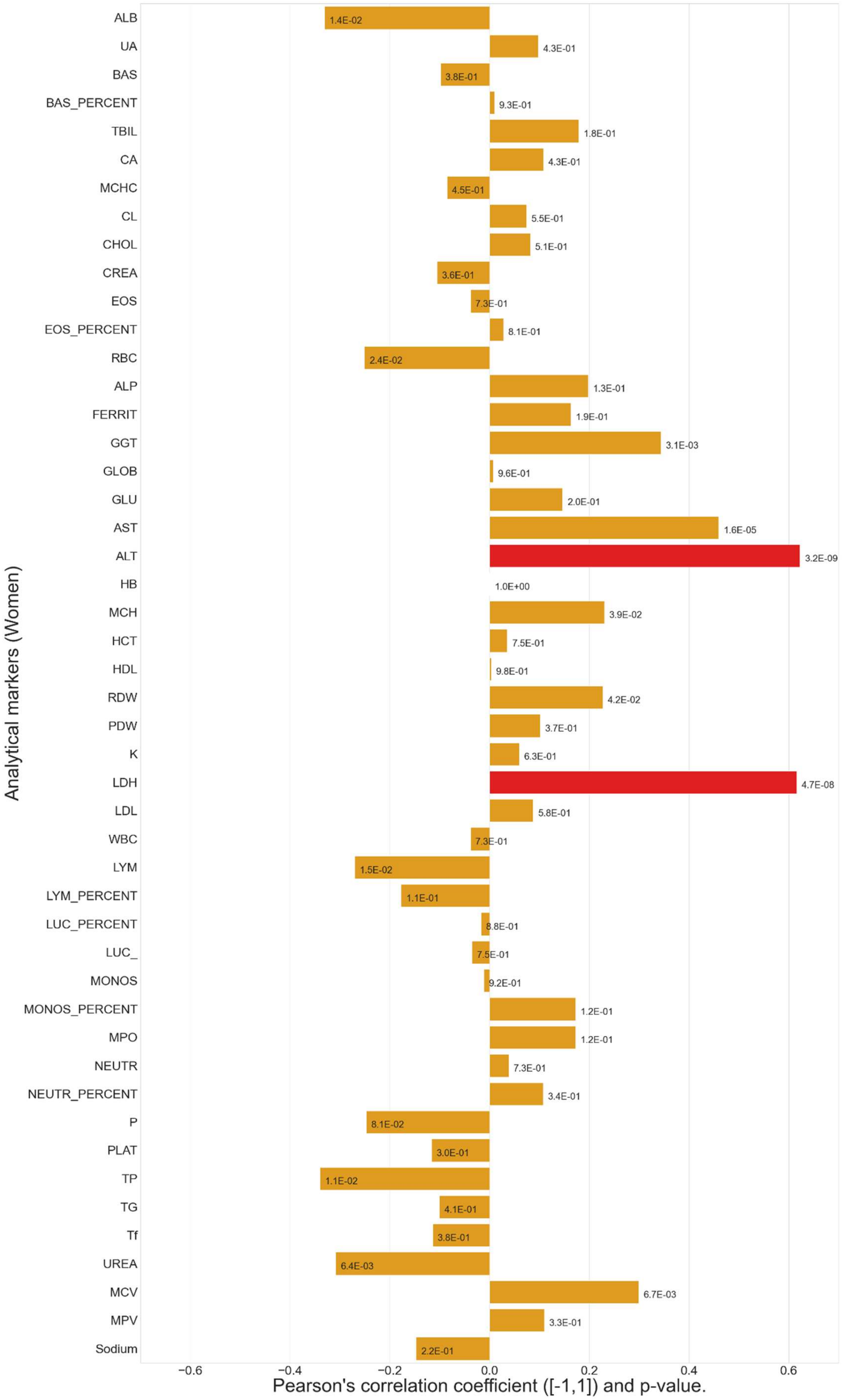

ALB 1.4E-02
UA 4.3E-01
BAS 3.8E-01
BAS_PERCENT 9.3E-01
TBIL 1.8E-01
CA 4.3E-01
MCHC 4.5E-01
CL 5.5E-01
CHOL 5.1E-01
CREA 3.6E-01
EOS 7.3E-01
EOS_PERCENT 8.1E-01
RBC 2.4E-02
ALP 1.3E-01
FERRIT 1.9E-01
GGT 3.1E-03
GLOB 9.6E-01
GLU 2.0E-01
AST 1.6E-05
ALT 3.2E-09
HB 1.0E+00
MCH 3.9E-02
HCT 7.5E-01
HDL 9.8E-01
RDW 4.2E-02
PDW 3.7E-01
K 6.3E-01
LDH 4.7E-08
LDL 5.8E-01
WBC 7.3E-01
LYM 1.5E-02
LYM_PERCENT 1.1E-01
LUC_PERCENT 8.8E-01
LUC_ 7.5E-01
MONOS 9.2E-01
MONOS_PERCENT 1.2E-01
MPO 1.2E-01
NEUTR 7.3E-01
NEUTR_PERCENT 3.4E-01
P 8.1E-02
PLAT 3.0E-01
TP 1.1E-02
TG 4.1E-01
Tf 3.8E-01
UREA 6.4E-03
MCV 6.7E-03
MPV 3.3E-01
Sodium 2.2E-01
Analytical markers (Women)
−0.6
−0.4
−0.2
0.0
0.2
0.4
0.6
Pearson's correlation coefficient ([-1,1]) and p-value.

Supplementary information: Figure S4. Rate of participants with alcohol consumption risk.

All cases **Men** **Women**

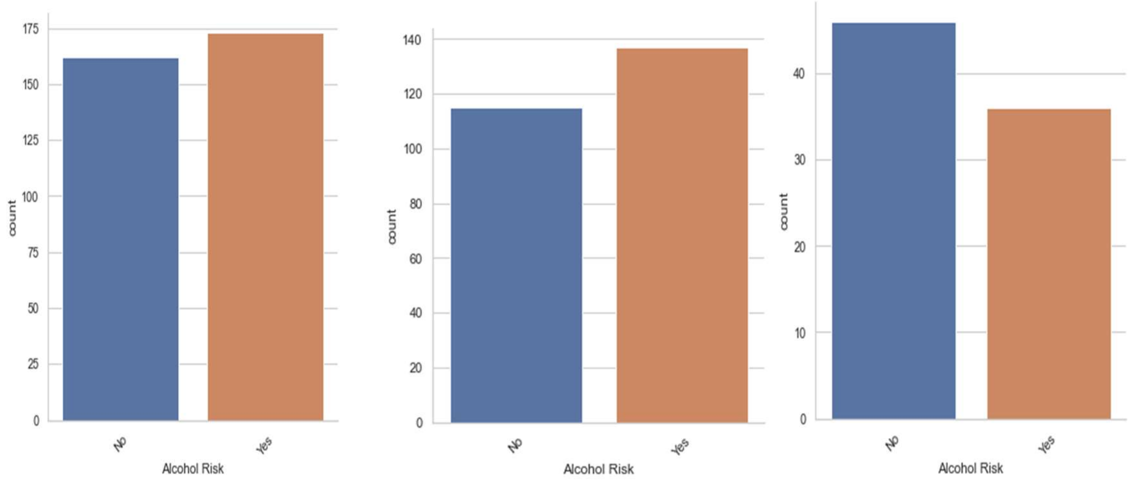

Supplementary information. Example S1. Example of manual use of the logistic regression model to make a prediction.

We take the case with ID = 1 and we will substitute the selected variables for the values presented by this patient, in the formula:

$Prob\ (Riesgo)$ = 1 / (1 + exp ($-Z$)) Prob (Risk) = 1 / (1 + exp (−Z)),

where Z is the sum of each variable multiplied by its corresponding coefficient. Since the training set was scaled, when predicting new cases, they must be scaled with the same scale learned (mean and deviation of each variable). So,

$$nuevo\_valorXi = (valorXi - mediaXi) / desviaciónXi$$

**Variables**	Patient value	Mean	Standard Deviation	Escaled
Mean Corpuscular Hemoglobin	31.20	31.055.589	2.445.915	0.059042
Gamma glutamyl transpeptidase	48.00	85.000.000	209.236.038	-0.176834
Red Blood Cells Dispersion Index	14.30	13.862.840	1.546.930	0.282598
Creatinine	0.97	0.903517	0.203253	0.327096
Total Bilirrubin	0.30	0.547737	0.472864	-0.523906
Mean Platelet Volume	9.20	9.470.393	1.169.194	-0.231264
Large Unstained Cells	0.20	0.183927	0.287767	0.055853
HDL (High Density Lipoprotein) - Cholesterol	36.00	59.043.716	17.241.018	-1.336.564

$$Z=0.6778+1.2997*0.059042-2.8804*0.176834+0.9826*0.282598+0.7294*0.327096-0.5458*0.523906-0.1743*0.231264+0.105*0.055853-0.7287*1.336564=1.01846$$

$$prob\text{ability(risk)}=1/(1+exp(-1.01846))=0.73467$$

As probability (risk)> 0.5 (standard threshold), risk is predicted. Which has been a true positive (TP) because the patient with ID = 1 is at risk.

In the case of trying to predict for a patient with some missing variable, that variable and its coefficient will be ignored in the calculation of Z (the imputation of missing values replaces it with the mean; and the scaling subtracts the mean, which leaves to 0).

Supplementary information. Example S2. Example of manual use for bayesian network for prediction.

We take the case with ID = 1 and we will substitute the selected variables for the values presented by this patient, in the formula:

Score (Risk) = $\prod P$ (Xi | Risk)

Score (No Risk) = $\prod P$ (Xi | No $Risk$)

$\forall Xi \in Variables_Selec$ted

And we return the prediction that maximizes the score result.

Taking as an example the patient with ID = 1, male with UBES_TOTAL_SEMANA = 36 (risk label):

Variables	Patient value
Study level	2
Basophils	0.04
Creatinine	0.97
Alkaline Phosphatase	101
Gamma glutamyl transpeptidase	48
Mean Corpuscular Hemoglobin	31.2
Hematocrit	50.5
Red Blood Cells Dispersion Index	14.3
Dehydrogen lactate	204
Urea	35

Thus, in this example the score for risk (which is the group to which our patient belongs) would be as follows:

Score (risk) = 0.000090 = 0.202 * 0.911 * 0.871 * 0.226 * 0.428 * 0.25 * 0.382 * 0.859 * 0.836 * 0.519.

If our patient were labeled without risk, the score would be as follows:

Score (no risk) = 0.000010 = 0.033 * 0.649 * 0.598 * 0.058 * 0.071 * 0.8 * 0.150 * 0.531 * 0.985 * 0.566.

The tag with the highest score is predicted. Since the score received for risk is higher, that is the prediction returned, which is a true positive achieved by the classifier.
